# The effects of statin therapy on aneurysm size, growth rate, and matrix metalloproteinases-9 levels in patients with aortic aneurysm: a systematic review and meta-analysis

**DOI:** 10.1186/s43044-023-00407-9

**Published:** 2023-10-13

**Authors:** Ketut Angga Aditya Putra Pramana, Yusra Pintaningrum, Basuki Rahmat

**Affiliations:** 1 General Practitioner of Mataram University, Mataram, Indonesia; 2 Interventional Cardiology Division, Cardiology and Vascular Department, Faculty of Medicine, Mataram University, Mataram, Indonesia

**Keywords:** Statin, Aortic aneurysm, Matrix metalloproteinase

## Abstract

**Background:**

Aortic aneurysm enlargement over time causes rupture, which frequently results in death. The family of proteases known as matrix metalloproteinases (MMP) is assumed to be proteolytic activity involved in the growth of aortic aneurysms. Statins are pleiotropic lipid-lowering medications with anti-inflammatory action. Statins can lower aneurysmal enlargement and MMP secretion, according to a number of studies, however the evidence is still up for debate. The purpose of this study is to assess how statins affect aortic aneurysm patient's aneurysm diameter size, growth rate, and MMP-9 levels.

**Methods:**

From January 2000 to December 2022, electronic journal searches in PubMed, ScienceDirect, and Cochrane were conducted to discover papers evaluating the effects of statin treatment in patients with aortic aneurysm. Aneurysm diameter size, growth rate, and MMP-9 levels were the outcomes we were looking for. Meta-analyses were run on the included studies, and mean differences (MD) and 95% CIs were calculated with Review Manager v5.4.

**Results:**

Our analysis includes a total of ten research. Statin medication substantially reduced aneurysm diameter size by 0.30 mm (*P* = 0.04; MD − 0.30; 95% CI − 0.58 to − 0.01) and growth rate by 0.34 mm/year (*P* < 0.00001; MD − 0.34; 95% CI − 0.40 to − 0.29) compared to placebo. There was no significant change in MMP-9 concentrations between individuals with aortic aneurysm who took a statin and those who did not.

**Conclusion:**

Overall, this meta-analysis demonstrates that statin medication is considerably helpful in reducing aneurysm diameter size and aneurysmal growth rate in individuals with aortic aneurysm.

## Background

In clinical practice, aortic aneurysms (AA) are increasingly commonly discovered. An AA is a persistent aortic dilatation that is more than three centimeters in diameter or 50% greater than usual [1]. The deterioration of arterial media and elastic tissues causes AA [2]. Upregulation of proteolytic pathways, apoptosis, oxidative stress, inflammation, and loss of the arterial wall matrix are some of the pathogenic mechanisms that contribute to the development of AA [3].

The prevalence of AA in elderly males over 60 years is 4–8%, while the incidence of newly diagnosed AAs is 3.5 to 6.5/1000 person years [2]. The risk of ruptured aneurysm (rAA) is directly connected to the aneurysm diameter, with a significant increase in risk when the aneurysm diameter surpasses 5.5 cm [4]. Even when patients make it to the hospital for surgical repair, rAA has one of the greatest fatality rates in all of medicine, not just in vascular surgery [5].

The development of aneurysms is linked to a persistent inflammatory response, a decrease in the number of smooth muscle cells, and an increase in the synthesis of matrix metalloproteinases (MMP) [4]. MMP are a group of extracellular enzymes that break down the cellular matrix, particularly the structural matrix proteins (elastin and interstitial collagens) [6]. There are naturally occurring MMP inhibitors known as tissue inhibitors of matrix metalloproteinases (TIMP), and an imbalance between MMP and TIMP inhibitors is thought to contribute to aneurysmal [7]. MMP-9 activity is an enzyme that has reportedly been crucial in the development of aneurysms [8]. MMP-9 plasma levels are elevated in the aneurysmal aortic tissue of AA disease compared to the normal aortic tissue [9, 10].

Hydroxymethylglutaryl-coenzyme A reductase inhibitors, also known as statins, are commonly used to decrease cholesterol [11]. However, due to their pleiotropic effects, statins have long been considered candidates to slow the rate of development and reduce the chance of AA rupture [12]. Statins have been shown in several trials to lower vascular inflammation and promote the regressive process of atherosclerotic plaque. It has been demonstrated that reducing vascular inflammation has a substantial role in the pathophysiologic collagenolytic pathway for the development of AA [13]. In addition, several studies showed that statins can reduce aneurysmal expansion and MMP secretion, but it remain debatable. The aim of this systematic review and meta-analysis are to evaluate the effects of statin on the aneurysm diameter size, growth rate, and MMP-9 levels in patients with aortic aneurysm.

## Methods

**Fig. 1:**
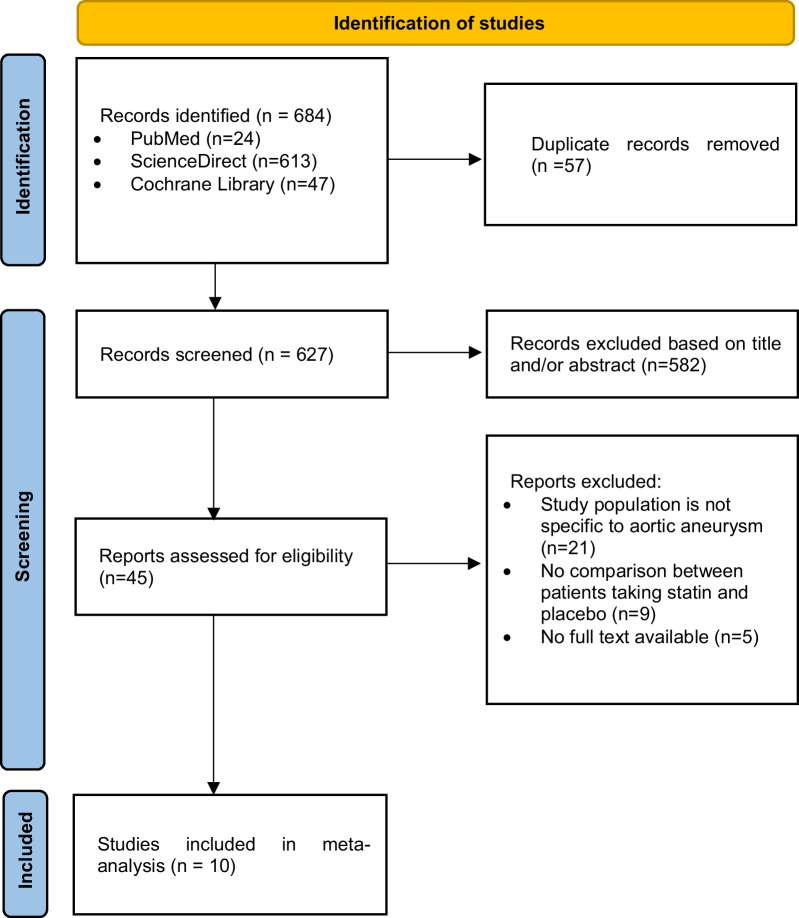
PRISMA flowchart showing results of the literature search

This systematic review and meta-analysis protocol's was entered with the ID CRD4202339508 into the International Prospective Register of Systematic Reviews (PROSPERO). The PRISMA 2020 Checklist and the PRISMA Preferred Reporting Items for Systematic Reviews and Meta-Analyses (PRISMA) extension for searching were used to conduct the study (Fig. 1).

### Data sources and search strategy

A systematic electronic search of PubMed, ScienceDirect, and Cochrane library was conducted from January 2000 to December 2022, for all randomized control trials (RCTs) and observational studies that assessing the effects of statin therapy in patients with aortic aneurysm. In addition, bibliographies of pertinent editorials and review articles were manually searched for potentially relevant articles. MeSH terms along with Boolean operators were used to produce a search strategy for each database. The following key search terms included: “Statin” and “Aortic Aneurysm.”

### Study selection process

After removing duplicate entries, KAAPP and BR independently reviewed first search records based on the title and abstract. After that, a separate complete text examination was carried out. The following criteria for studies were used for inclusion: (1) Comparison of the results of statin- therapy with placebo in the patients with aortic aneurysm; (2) English-language study analyses. Consensus was reached in resolving disagreements, including those of the other author (YP).

### Outcome measures

The outcome of interest of this meta-analysis is aneurysm diameter size, growth rate, and MMP-9 levels.

### Data extraction and quality assessment

The baseline parameters of included studies were retrieved by KAAPP and BR. The chosen Newcastle-Ottawa Scale (NOS) for observational studies was then used by KAAPP and BR to conduct a comprehensive quality assessment of the included research [14]. The quality of investigations was rated as poor (5 points), moderate (5–7 points), or good (> 7 points). Any differences were settled by consensus, incorporating the other author's viewpoint (YP).

### Statistical analysis

The Mantel–Haenszel fixed-effects models using mean difference (MD) as the effect measure and the corresponding 95% confidence interval (CI) were used to pool the data. The Higgins *I*^2^ statistic was used to gauge statistical heterogeneity between groups. In particular, an *I*^2^ = 0 meant that there was no heterogeneity, and we defined substantial heterogeneity as *I*^2^ values exceeding 50%. Given the study heterogeneity, we utilized the random-effect models to compute the pooled mean difference of survival rates of aortic aneurysm size and MMP-9 levels in patients with aortic aneurysm. We used Egger’s test funnel plot to assess the potential publication bias for this study. For all analyses, Review Manager 5.4.1 was employed. Statistical significance was defined as a two-sided p value of 0.05 or less.

## Results

### Study search and selection results

The first search method produced 684 entries. Ten articles were included in the systematic review and meta-analysis after a complete text analysis of 45 potentially relevant records (Fig. 1).

### Baseline characteristics and quality assessment

The included studies, characteristic baselines are presented in Table 1. All studies were cohort studies and the authors did not find RCT studies suitable for inclusion in this systematic review and meta-analysis. Among the 10 included studies, the total patients of included studies were 40,496 patients, 26,910 patients received statin therapy and 13,586 patients received placebo.

**Table 1:** Main characteristics of the studies included in the meta-analysis

References	Country	Design	Sample size		Follow up (months)	Outcome
			Statin	Placebo		
Abisi et al. [8]	UK	Cohort	21	61	1	MMP-9 levels
Evans et al. [15]	UK	Cohort	7	10	1	MMP-9 levels
Hurks et al. [16]	Netherlands	Cohort	135	81	1	MMP-9 levels
Karrowni et al. [2]	US	Cohort	136	75	12	Growth rate
Mosorin et al. [5]	Finland	Cohort	34	87	42	Growth rate
O’Donell et al. [17]	US	Cohort	25,997	11,941	36	Aneurysm size
Raux et al. [18]	France	Cohort	120	46	24	Aneurysm size
Stein et al. [19]	US	Cohort	368	1184	NR	Aneurysm size and growth rate
Sukhija et al. [20]	US	Cohort	75	55	24	Aneurysm size
Wilson et al. [21]	UK	Cohort	17	46	NR	MMP-9 levels

**Fig. 2:**
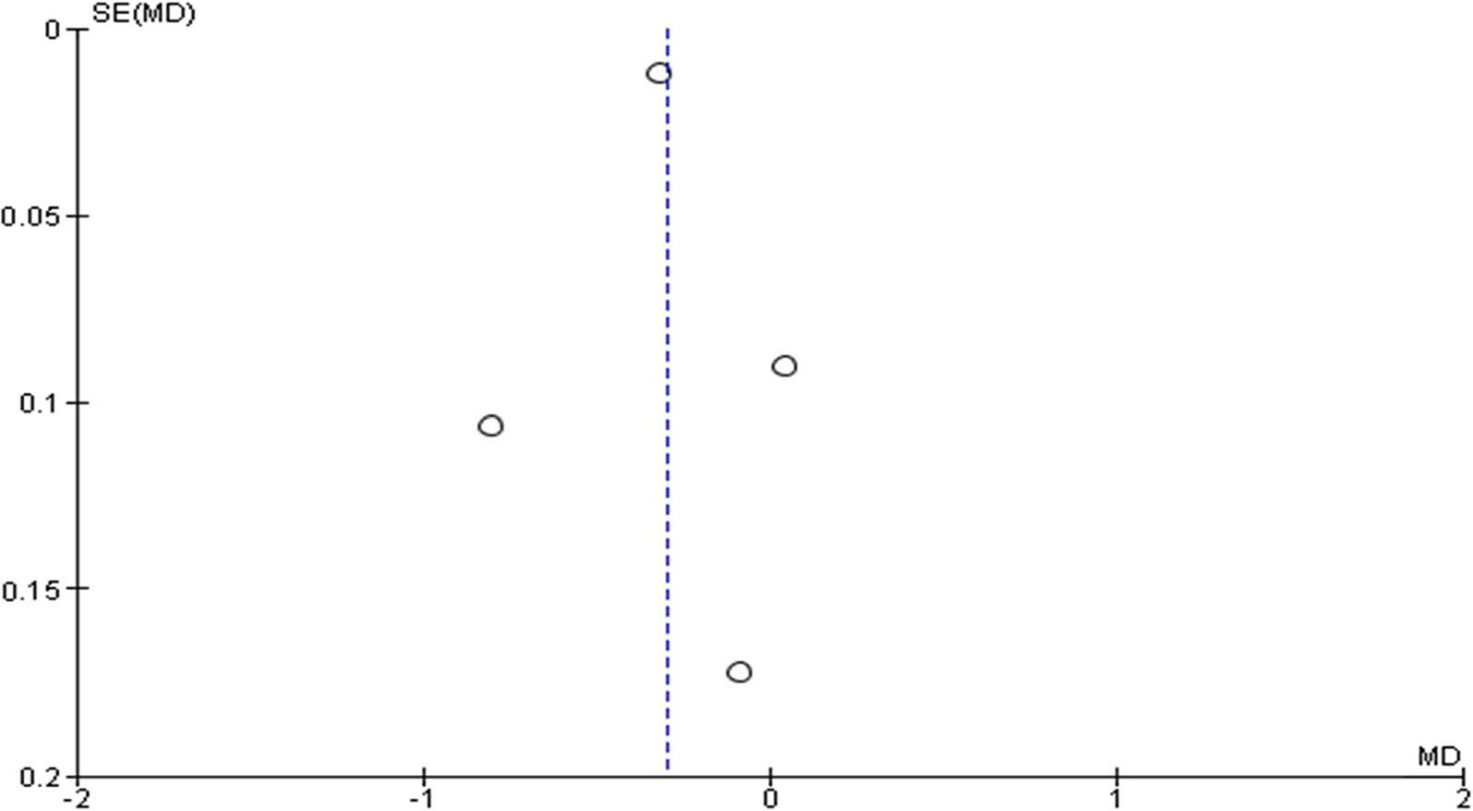
Funnel plot of the effect of statin therapy on aneurysm size of aortic aneurysm

**Fig. 3:**
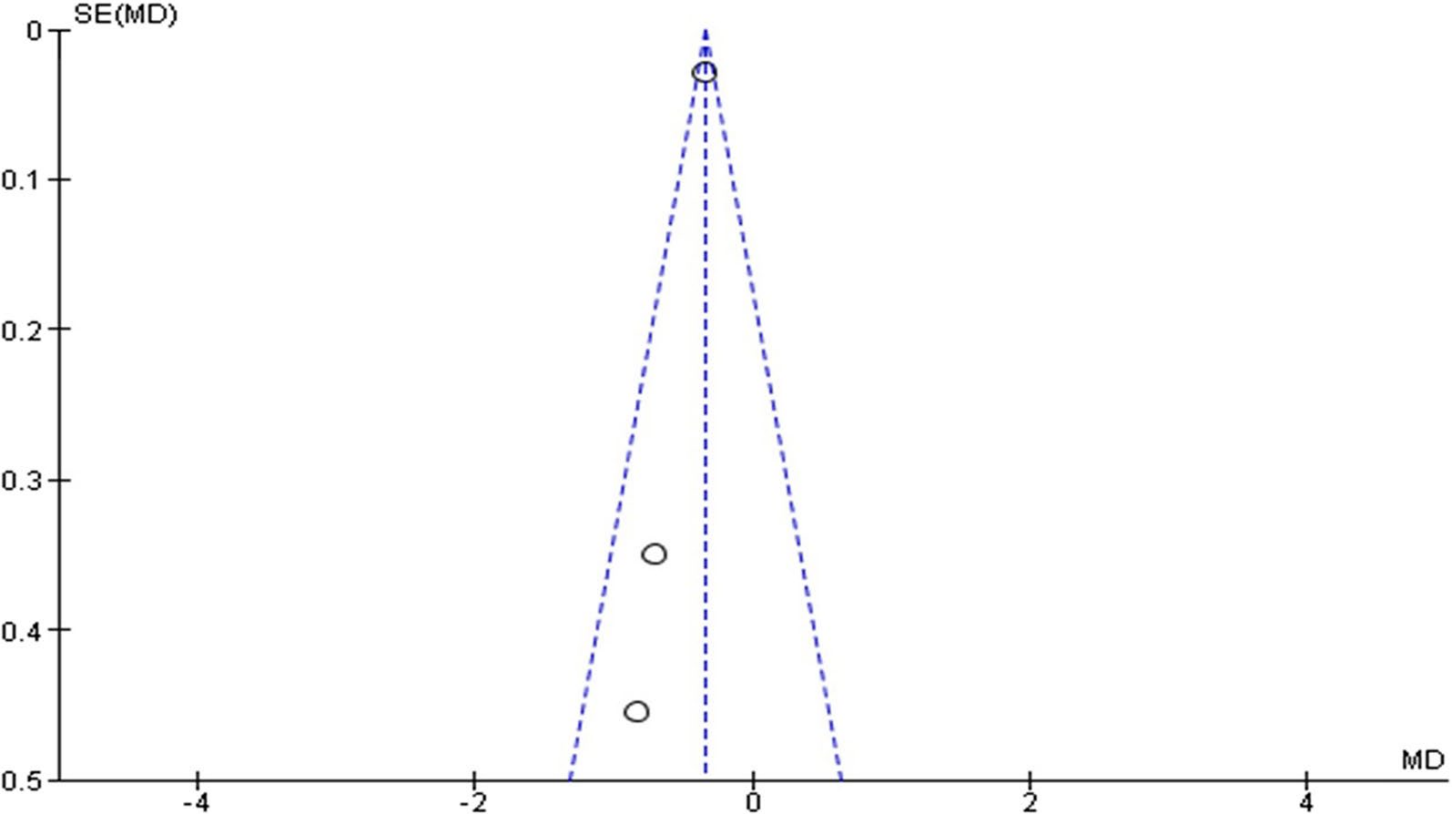
Funnel plot of the effect of statin therapy on growth rate of aortic aneurysm

**Fig. 4:**
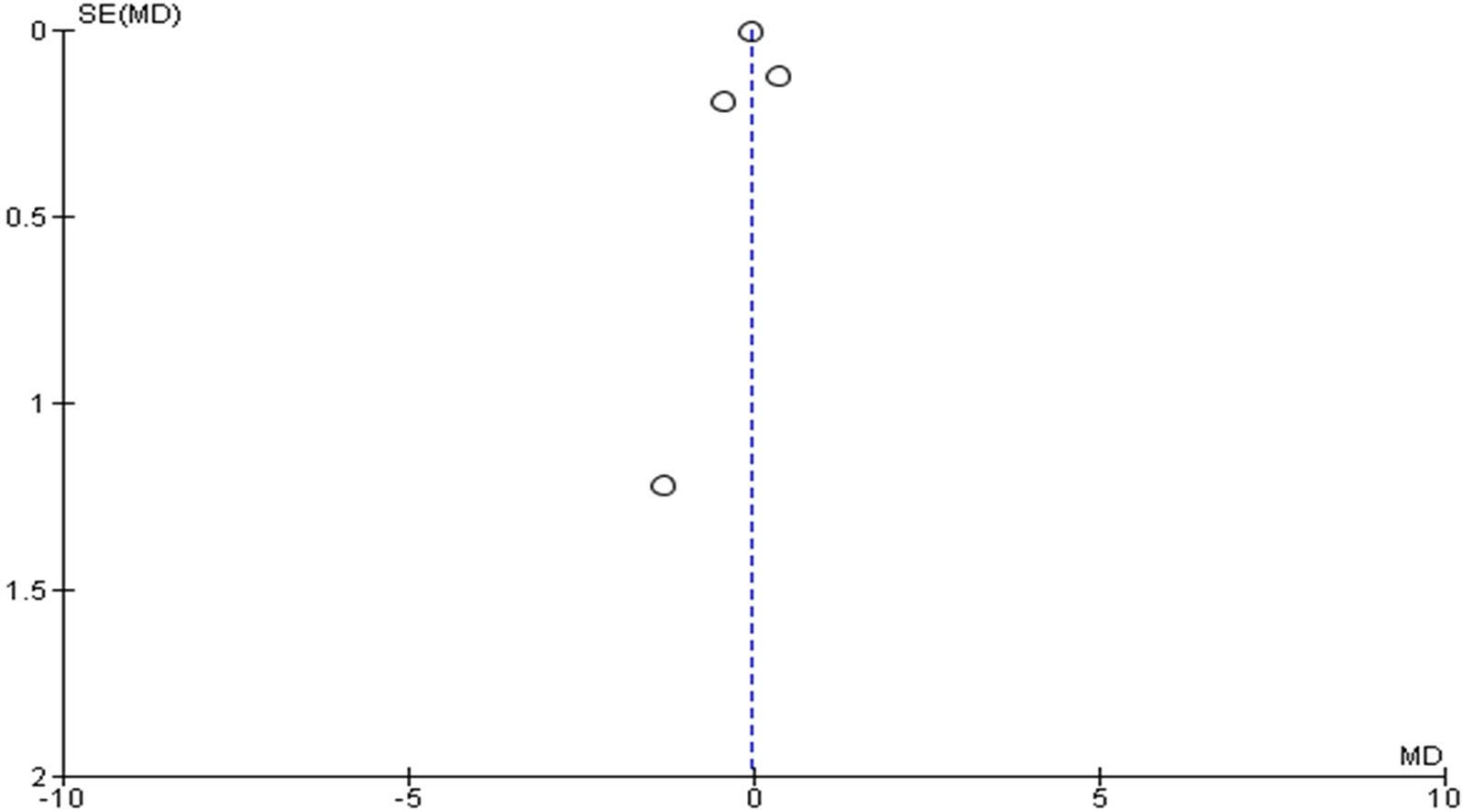
Funnel plot of the effect of statin therapy on MMP-9 levels in patients with aortic aneurysm

In ten studies, quality and bias risk were evaluated using the NewCastle–Ottawa Scale (Table 2). All observational studies ranked their overall risk of bias as moderate, and some were classified as high-quality research. No observational research has a significant bias risk. Based on analysis of the funnel plot in Figs. 2, 3, and 4, publication bias of the papers that were included cannot be excluded.

**Table 2:** Quality assessment of included studies based on Newcastle–Ottawa Scale

References	Country	Selection	Comparability	Outcome	Total
Abisi et al. [8]	UK	***	**	**	7
Evans et al. [15]	UK	***	**	**	7
Hurks et al. [16]	Netherlands	****	**	***	9
Karrowni et al. [2]	US	***	*	**	6
Mosorin et al. [5]	Finland	***	**	**	7
O’Donell et al. [17]	US	****	**	***	9
Raux et al. [18]	France	****	*	**	7
Stein et al. [19]	US	**	**	*	5
Sukhija et al. [20]	US	***	*	**	6
Wilson et al. [21]	UK	**	**	*	5

### Pooled analyses for clinical outcomes

#### Effect of statin therapy on aneurysm diameter size

**Fig. 5:**
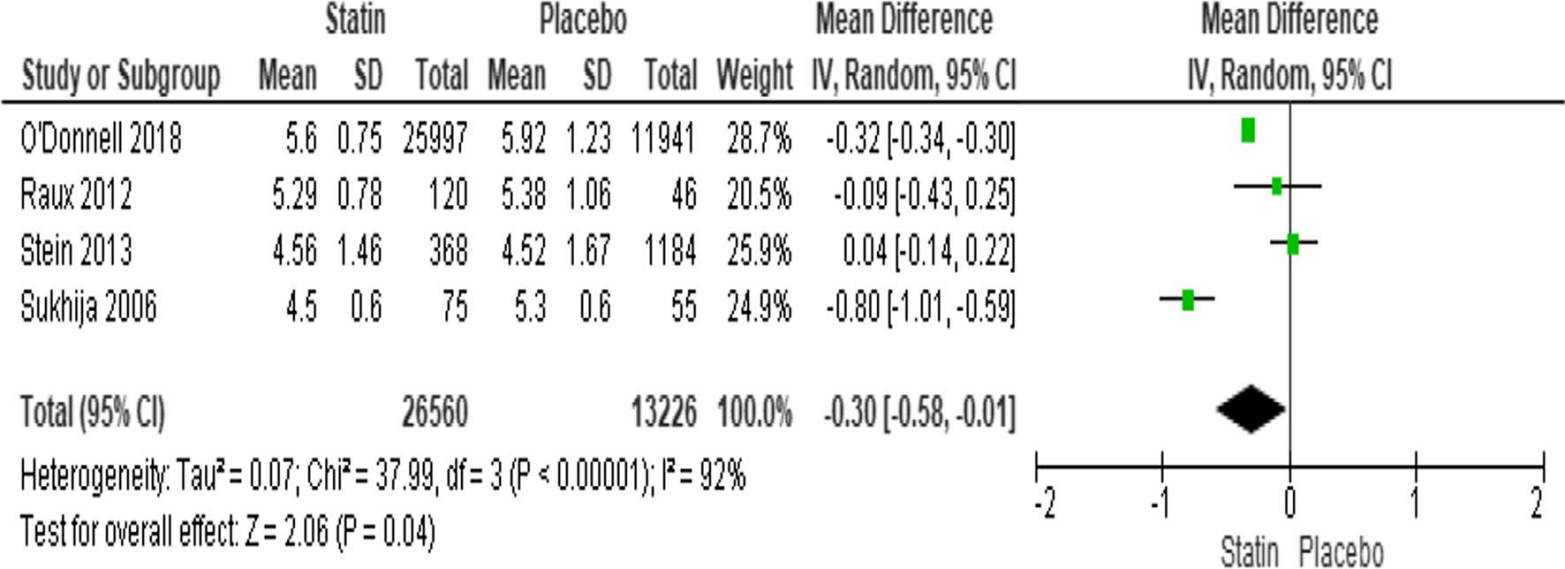
Forest plot of the effect of statin therapy on aneurysm size of aortic aneurysm

The aneurysm diameter size was reported in four trials. They were subjected to a scan for considerable heterogeneity (*I*^2^ = 92%; heterogeneity *P* 0.00001; Fig. 5). Statins substantially lower aneurysm diameter by 0.30 mm (*P* = 0.04; MD − 0.30; 95% CI − 0.58 to − 0.01) when compared to placebo, according to the results of a random effect model. The Egger’s test funnel plot of the included study is presented in Fig. 2. Based on the result, we concluded that there was a potential publication bias for this meta-analysis study (asymmetry of the scatter plot in the triangle).

#### Effect of statin therapy on growth rate

**Fig. 6:**
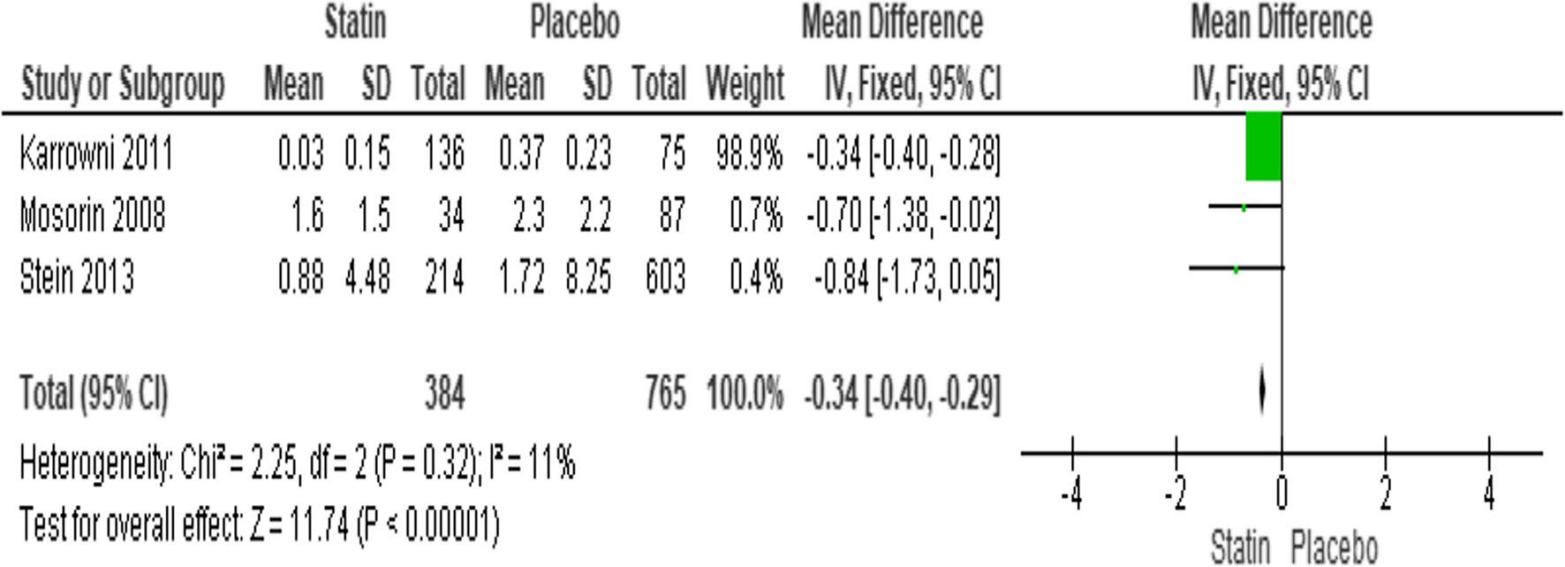
Forest plot of the effect of statin therapy on growth rate of aortic aneurysm

The growth rate was reported by three investigations. They were examined for non-significant heterogeneity (*I*^2^ = 11%; heterogeneity *P* = 0.32; Fig. 6). Statins substantially slow growth rate by 0.34 mm/year (*P* < 0.00001; MD − 0.34; 95%CI − 0.40 to − 0.29) when compared to placebo, according to the results of a fixed effect model. The Egger’s test funnel plot of the included study is presented in Fig. 3. Based on the result, we concluded that there was a potential publication bias for this meta-analysis study (asymmetry of the scatter plot in the triangle).

#### Effect of statin therapy on MMP-9 levels

**Fig. 7:**
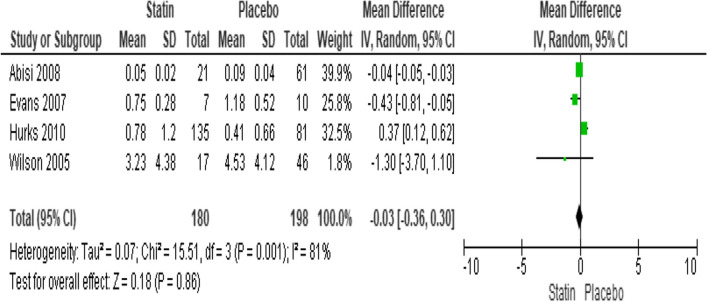
Forest plot of the effect of statin therapy on MMP-9 levels in patients with aortic aneurysm

The MMP-9 values were reported by four investigations. They had a high amount of heterogeneity (*I*^2^ = 81%; heterogeneity *P* = 0.001; Fig. 7) scanned. The results of the random effect model showed that statins did not substantially lower MMP-9 levels when compared to a placebo (*P* = 0.86; MD − 0.03; 95% CI − 0.36 to 0.30). The Egger’s test funnel plot of the included study is presented in Fig. 4. Based on the result, we concluded that there was a potential publication bias for this meta-analysis study (asymmetry of the scatter plot in the triangle).

## Discussion

The final systematic review and meta-analysis included ten studies with a combined total of 40,496 patients. Statin treatment was associated with a significantly decreased aneurysm diameter size and growth rate compared to placebo. In individuals with aortic aneurysms using a statin versus not taking one, there was no discernible variation in the concentration of MMP-9 levels.

Because the pathophysiology of AA development is complicated and incompletely understood, there are currently no viable medicinal treatments. A persistent inflammatory response, a decrease in the number of smooth muscle cells, and an excess of MMP synthesis are all linked to aneurysm development [22]. Numerous inflammatory infiltrates including macrophages and lymphocytes have been seen in both the media and adventitia in studies of human AA tissue [23]. Different proteinases are secreted by activated macrophages, which causes an imbalance in the production and breakdown of connective tissue proteins. The human aorta wall is destroyed by a number of extracellular proteinases, and MMP-9 in particular has generated attention in this process [24, 25].

Statin medication effectively reduced the size of the aneurysm and slowed the growing rate of AA when compared to the placebo group. Although the precise mechanism by which statins may slow the progression of AA is still unclear, there are a number of ideas. Statins have a number of pleiotropic effects that focus on the pathophysiologic processes involved in the genesis of AA. Through its anti-inflammatory, anti-oxidative, protease-inhibiting, and up-regulation of the production of extracellular matrix proteins, they are anticipated to prevent the development of AA [26]. Statins block a number of important molecules, such as MMP generated by macrophages, as well as several inflammatory mediators [27]. Protein isoprenylation, which is important in the signaling pathways that cause inflammation, is thought to be affected, which is one explanation for these consequences [28].

According to a study by Kalyanasundaram et al., statins preserve elastic protein and vascular smooth muscle cells while also drastically reducing the protein levels of MMP and inhibiting the growth of experimental aneurysms [22]. According to a different research by Hirotaka et al., statin directly regulated the biology of the AA wall and prevented the synthesis of MMP in the AA wall by preventing neutrophil and macrophage activation [27]. Other experimental investigations have indicated that statins have a variety of other favorable benefits, including improved endothelial function via an increase in endothelial nitric oxide synthase, inhibition of medial VSMC death, and a decrease in macrophage infiltration into the vascular wall [29, 30].

To the best of the authors' knowledge, this is the first meta-analysis to look at the impact of statins on plasma MMP-9 levels in aortic aneurysm patients. Our investigation found no significant changes in MMP-9 levels or activity in AA between statin and placebo. The findings of this study are corroborated by other investigations by Hurks et al. and Evans et al. that found no significant changes in MMP-9 levels [15, 16]. Likely explanations for our findings lack of statistical significance because of differences in study populations of included studies, short follow-up period, small sample size, and possible relevance of other types of proteases (like other types of MMPs or TIMPs) that involved in the pathogenesis of AA.

Despite the fact that our findings appear to be genuine and consistent with previous research, they should be viewed in light of certain limitations. Firstly, our study solely included cohort studies. As a result, there is the possibility of information dissemination and confusing bias. Second, despite the fact that we meticulously searched the available publications, used specified research inclusion criteria, and strictly completed data collection and analysis, there was substantial heterogeneity across included studies. Third, Although the results of our meta-analysis show several significant variables, further research is needed, especially RCT studies or systematic review and meta-analysis which includes RCT studies regarding the role of statin therapy in aortic aneurysms to establish a causal relationship.

## Conclusions

In conclusion, this systematic review and meta-analysis represents a contemporary evaluation of best available evidence on the association between statin therapy and aortic aneurysm. Our analysis demonstrated that statin therapy significantly effective on reduce the aneurysm diameter size and aneurysmal growth rate in patients with aortic aneurysm. Based on the results of our meta-analysis, further research is needed, especially RCT studies or systematic review and meta-analysis which includes RCT studies to determine whether statin therapy can later be used as routine therapy in patients with aortic aneurysms

## Data Availability

Not applicable.
